# Status Lymphaticus; Its Relation to Sudden Death1Read before the Surgical Section of the Buffalo Academy of Medicine, September 6, 1904.

**Published:** 1904-11

**Authors:** Edgar R. McGuire

**Affiliations:** Buffalo, N. Y.; Assistant to the Surgical Clinic of Buffalo General Hospital.


### Status Lymphaticus; Its Relation to Sudden Death.[1]

SYNONYMS; LYMPHATIC CONSTITUTION, LYMPHATISM, STATUS THYMACUS.

By EDGAR R. McGUIRE, M. D., Buffalo, N. Y.
Assistant to the Surgical Clinic of Buffalo General Hospital.

THE study of this rather obscure condition is of very great interest to the physician and surgeon because it throws some light on a hitherto hidden field, the causation of many sudden deaths. Hence it seems proper to epitomise the surgical aspects of this condition, more especially in its relation to fatal collapse from anesthetics and other surgical conditions.

For many years enlargement of the thymus gland has been noted at autopsies, but the relation of this enlargement to other accessory features, which go to make up status lymphaticus, was unknown until Rokitausky in 1842, first recognised the condition in its entirety. Naturally, it was at first confused with lymphatic tuberculosis, then known as scrofula.

Normally, the thymus gland begins involution shortly after birth, and the process should be completed at puberty. In status lymphaticus this involution does not occur, but the gland continues to enlarge into and even beyond adult life. This enlargement varies greatly in different cases, ranging from 20 to 135 grams.

The pathology of this condition is rather obscure. The thymus rarely shows anything abnormal other than the enlargement. Occasionally an acute hyperemia is present, and at times from the cut gland a milky fluid exudes. There is, however, general hyperplasia of the lymphatic system. Both the superficial and deep nodes are enlarged, especially the cervical, axillary, inguinal, mesenteric and retroperitoneal. The spleen is enlarged and hyperemic. The Malpighian bodies are packed with lymphoid cells, and occasionally, the pulp becomes infiltrated with them. Not infrequently the heart and arteries share in this general lymphoid infiltration. Because of this feature, Virchow originated the term lymphatic-chlorotic-constitution. The cause of the narrowing of the aorta, frequently seen at autopsy, has long been a matter of doubt, but it is now thought by some to be due to this lymphoid infiltration.

While as yet it would be going too far afield to ascribe all cases of rickets to status lymphaticus, nevertheless, it is an interesting fact that the majority of the cases of status lymphaticus

1. Read before the Surgical Section of the Buffalo Academy of Medicine, September 6, 1904.

show marked evidences of rickets. Beyond question there is some unknown relation between the two and this may explain the benefit that is said to occur in rickets from the administration of the extract of thymus gland.

Furthermore there seem to be some peculiar relation between the thyroid and the thymus glands. In about one half the cases of enlarged thymus, the thyroid is found enlarged. Experimentally when either gland is removed, enlargement of the other occurs. The theory of internal secretions is here an inviting explanation. It is also well within the realm of reason to assume that the loss in secretion incurred by the removal of one gland causes a compensatory hypertrophy in the other.

Clinically, there are a number of conditions which bear a striking resemblance to status lymphaticus.

Thymic asthma or, as it is sometimes called, laryngismus stridulus comes in this category. Here occasionally an absorbingly interesting medico-legal question may arise, for while death in such cases usually occurs only after definite warning, yet it may, and sometimes does, occur instantly, giving rise to suspicion of foul play regarding the cause of death.

A lively controversy has arisen regarding the real cause of death in these cases of thymic asthma. One explanation is that death occurs by mechanical pressure of the enlarged thymus on the trachea and large vessels, while another claims death is due to sudden arrest of the heart's action by reflex cardiac paralysis. Quite probably both are in the main correct, as it is most likely that death does not occur in every instance from the same immediate cause.

Convulsions in infants may arise from so many causes it seems unwise to add others to a now over-burdened list. However, as our information grows from autopsy reports we find status lymphaticus plays a rather important rôle. So much confirmatory evidence is now at hand, that, in convulsions of any kind in infants, the question of status lymphaticus should be considered, and until it can be eliminated a very guarded prognosis should be given.

Status lymphaticus in its relation to sudden death from surgical narcosis is of particular interest to the surgeon. So many factors enter into a discussion of this form of death, that it is very difficult to draw positive conclusions. Probably most of these deaths, much as we dislike to admit it, are due directly to the anesthetist. By this I mean, that in any given death from anesthesia, if we had an anesthetist sufficiently skilled, the death might usually have been prevented. However, there are many cases that cannot be charged to the anesthetist, and among them are those of status lymphaticus. Most of the evidence regarding these cases comes from Austria and Germany where thorough autopsies have been performed with a view to determining the relationship of this condition to sudden death during narcosis. In these countries, chloroform is used almost exclusively; hence it has been assumed by many that chloroform is particularly dangerous in this condition. Recent investigations, however, seem to show that ether is equally as dangerous under similar circumstances. Death may occur at any stage of anesthesia and it has been known to occur some minutes after the anesthetic has been stopped. A rather significant fact is that the majority of such deaths occur during operations for adenoids and enlarged tonsils; those conditions being among the most prominent manifestations of status lymphaticus.

The literature of status lymphaticus in its relation to sudden deaths makes very interesting reading, because of the peculiarity of the cases reported. Nordman cites several cases which died suddenly, either in the water or shortly after coming to the shore. In each case the cause of death was confirmed by autopsy and none of them showed any of the usual signs of death by drowning. The case of Professor Langerhans' son, of Berlin, is also interesting. He died immediately following the injection of antitoxin for diphtheria, and consequently the death was ascribed at first to the antitoxin. A very wide spread discussion followed and from the autopsy findings the pathologists concluded that status lymphaticus was the cause of death.

That all these cases die suddenly is certain, but just why they should live comfortably for years and then unexpectedly succumb to such apparently trifling causes is, indeed, difficult to comprehend.

The diagnosis of status lymphaticus is particularly difficult. There are, however, many suggestive features which when present should lead to a well-marked suspicion of this condition. In the first place the close relationship existing between this condition and rickets, gives us an important lead. In the majority of these cases, evidences of rickets are present. There will usually be found enlargement of the cervical, axillary and inguinal lymphatics, and it may be even possible to palpate enlarged mesenteric nodes. Adenoid growths in the naso-pharynx, enlargement of the spleen together with an area of dulness over the thymus complete the clinical picture. Individually, these signs mean but little, but when present together, a tentative diagnosis of status lymphaticus would be well founded.

The etiology of this condition is equally obscure. Many believe that as our information increases, rickets and status lymphaticus will be invariably associated. At the present time rickets is quite generally believed to be an infection by a variety of organisms. The lymphatic enlargement in status lymphaticus points toward an infection of some kind.

Clinically, every surgeon occasionally meets with cases of sudden death which are particularly difficult to explain. An autopsy will apparently reveal little or nothing to account for death. A few such cases have occurred in Dr. Park's service several hours after operation where autopsy revealed absolutely nothing to account for the death. The question naturally arises: How much has the lymphatic state to do with such cases? Certainly in all autopsies upon cases of sudden death very careful investigation should be made of the thymus and the whole lymphatic system.

*Treatment.*—In those cases of severe dyspnea where the probable cause is an enlarged thymus, surgical treatment should be immediate. Two such successful cases are reported. One by König, where a portion of the thymus was removed and the remainder anchored by sutures to the fascia of the sternum; another by Siegel, where tracteotomy was performed with no relief, until a long canula was passed well beyond the enlarged thymus, when the relief was immediate. In the latter case the thymus was also sutured to the sternum. Both cases recovered with no return of dyspnea. Where evidences of rickets are present suitable medical treatment should be prescribed.

Simply because we have an enlarged thymus, it is not necessarily certain that we are having an abundance of thymus secretion, hence in some of these cases extract of thymus gland has given some relief. On the other hand, where this thymal secretion is present in abundance adrenalin would seem to be indicated theoretically.

The most striking results, however, obtain after removal of growths from the naso-pharynx. While due allowance in the improvement must be made for the removal of the mechanical obstruction, yet the remarkable change in the facial expression, together with the improved intellect suggests a more widespread influence.

### LITERATURE.

Ewing, New York Med. Journal. July 10, 1897.
Norton, Phil. Med. Journal. 1898, p. 249.
Hinkel, New York Med. Jour. October 29, 1898.
Olmacher, Bulletin Ohio Hospital for Epileptics, Nos. 1, 2, 3. 1898-99.
Halstead, Phil. Med. Journal. 1900, p. 859.
Blake, Annals of Surgery. 1902.
Griffin, American Medicine. 1903, pp. 989-94.
Dudjean, British Medical Journal. 1903, 11, 1533.
Koppel, Albany Medical Annals. 1903, XXIV., pp. 430-33.
Blumer, Pediatrics. 1904, XVI., pp. 22-46.

1175 Main Street.

